# Neural plasticity in hypocretin neurons: the basis of hypocretinergic regulation of physiological and behavioral functions in animals

**DOI:** 10.3389/fnsys.2015.00142

**Published:** 2015-10-21

**Authors:** Xiao-Bing Gao, Gretchen Hermes

**Affiliations:** ^1^Section of Comparative Medicine, Yale University School of MedicineNew Haven, CT, USA; ^2^Program on Integrative Cell Signaling and Neurobiology of Metabolism (ICSNM), Yale University School of MedicineNew Haven, CT, USA; ^3^Department of Psychiatry, Yale University School of MedicineNew Haven, CT, USA

**Keywords:** hypocretin/orexin, perifornical/lateral hypothalamus, energy balance, sleep-wake homeostasis, drug addiction

## Abstract

The neuronal system that resides in the perifornical and lateral hypothalamus (Pf/LH) and synthesizes the neuropeptide hypocretin/orexin participates in critical brain functions across species from fish to human. The hypocretin system regulates neural activity responsible for daily functions (such as sleep/wake homeostasis, energy balance, appetite, etc.) and long-term behavioral changes (such as reward seeking and addiction, stress response, etc.) in animals. The most recent evidence suggests that the hypocretin system undergoes substantial plastic changes in response to both daily fluctuations (such as food intake and sleep-wake regulation) and long-term changes (such as cocaine seeking) in neuronal activity in the brain. The understanding of these changes in the hypocretin system is essential in addressing the role of the hypocretin system in normal physiological functions and pathological conditions in animals and humans. In this review, the evidence demonstrating that neural plasticity occurs in hypocretin-containing neurons in the Pf/LH will be presented and possible physiological, behavioral, and mental health implications of these findings will be discussed.

## Introduction

In order to ensure their own survival and that of their species, animals need to intake and use energy, respond to stimuli, adapt to their environments and generate offspring. The hypothalamus has been demonstrated as a critical area of the brain that regulates these functions. As early as the first half of the 20th century, von Economo (1930) described that the disruption of the posterior hypothalamus led to sleepiness and coma in human patients. Later, Anand and Brobeck (1951) reported that a lesion in the lateral hypothalamus inhibited food intake in rodents. In the meanwhile Olds and Milner (1954) reported that the lateral hypothalamus was one of the “reward seeking” areas in the brain, in which electric stimulations led to positive reinforcement in rodents. These groundbreaking works laid the foundation for a contemporary understanding of the hypothalamus. While several prominent neuronal systems in the hypothalamus have been well established for decades, the hypocretin system was discovered less than two decades ago. Nevertheless, elucidating the role of this system in the regulation of brain functions, such as the sleep/wake cycle, appetite, reward and stress response, has re-shaped the understanding of the neural processes underlying complex behaviors in animals as well as neurological and psychiatric conditions in humans in an unprecedented way.

The neuropeptide hypocretin (orexin) was discovered by two groups of researchers independently (de Lecea et al., 1998; Sakurai et al., 1998). As early as 1996 many mRNA species that are only expressed in the hypothalamus of rats were identified by using directional tag PCR subtraction, among which a novel clone named “clone 35” was demonstrated to encode a pre-prohormone (Gautvik et al., 1996). Later, two novel peptidergic hormones named hypocretin 1 and 2, selectively synthesized in neurons in the perifornical and lateral hypothalamus (Pf/LH), were found to be the products of this pre-prohormone through proteolytic cleavage (de Lecea et al., 1998). At the same time, two G-protein coupled receptors and their natural ligands that are exclusively expressed in the lateral hypothalamus were reported by another research group (Sakurai et al., 1998). Since these molecules could promote food intake in animals, they were named as orexins (orexin-a and orexin-b) (Sakurai et al., 1998). Eventually hypocretin and orexin were demonstrated to be the same peptidergic hormone.

It is now clear that at the cellular level hypocretin enhances synaptic transmission and elevates intracellular calcium levels in various neuronal types in the brain (van den Pol et al., 1998; Hagan et al., 1999; Davis et al., 2003; Korotkova et al., 2003). Since its discovery, the hypocretin system has emerged as a vital component of the brain circuitry governing basic animal behaviors and higher functions. Based on the wide distributions of hypocretin-containing nerve fibers and hypocretin receptors in the central nervous system (CNS) and the roles of hypocretin in animal behaviors ranging from homeostatic regulation to cognitive functions (Peyron et al., 1998), many competing hypotheses have been proposed to characterize the general role played by this system in the CNS (Chase, 2013; Gao and Horvath, 2014; Mahler et al., 2014; Sakurai, 2014). However, a growing body of evidence from our laboratory and others indicates that a true understanding of the functions of the hypocretin system requires a detailed characterization of the activity and plasticity in hypocretin neurons under various physiological and pathological conditions.

## Basic Synaptic Organization of the Hypocretin System

Hypocretin neurons possess many unique characteristics when compared to other neuronal types in the brain. Firstly, by using whole-cell patch clamp recordings and electron microscopic investigations, Horvath and Gao (2005) and others revealed that the frequency of miniature excitatory postsynaptic currents (mEPSCs) recorded at the soma of hypocretin cells was about 10-fold higher than that of miniature inhibitory postsynaptic currents (mIPSCs) and that there were more asymmetric (putatively excitatory) synapses than symmetric (putatively inhibitory) synapses on cell bodies of hypocretin neurons (Horvath and Gao, 2005; Xie et al., 2006). These results strongly indicate that the cell bodies of hypocretin neurons are predominately under the innervation of excitatory (glutamatergic) compared to inhibitory (GABAergic) synapses. This unique synaptic architecture is substantially distinct from other long projection neurons such as pyramidal neurons in the neocortex, in which mostly inhibitory (GABAergic) synapses exist on the somata of cells (Douglas et al., 2004). The unique arrangement of excitatory and inhibitory synapses on hypocretin neurons is consistent with the finding that the blockade of ionotropic glutamategic transmission onto hypocretin neurons with selective glutamatergic antagonists significantly attenuates the generation of action potentials. Conversely, the inhibition of GABA_A_–mediated neurotransmission has no significant effects on spontaneous action potential firing in these neurons (Li et al., 2002; Xie et al., 2006). The origin of the nerve fibers that synapse on hypocretin neurons is not well established but they may be from neuronal systems both within and beyond the Pf/LH area (Li et al., 2002; Henny and Jones, 2006a,b; Yoshida et al., 2006).

Secondly, glutamatergic synapses on hypocretin neurons are distinctive as well. The ratio between AMPA receptor (AMPAR)-mediated excitatory postsynaptic currents (EPSCs) and NMDA receptor (NMDAR)-mediated EPSCs (AMPAR/NMDAR ratio) is a parameter used to examine the existence of “silent” synapses on neurons (Perkel and Nicoll, 1993; Isaac et al., 1995). The AMPAR/NMDAR ratio is less than one under basal conditions in CA1 pyramidal neurons in the hippocampus and dopamine (DA) neurons in the ventral tegmental area (VTA) in young adult animals (Isaac et al., 1995; Ungless et al., 2001). It has been shown by us that the AMPAR-mediated EPSC is significantly larger (>2 fold) than that mediated by NMDARs under the baseline condition (the AMPAR/NMDAR ratio is larger than 1.0) in hypocretin neurons at the similar developmental stage (Rao et al., 2007, 2008, 2013). Since the existence of a significant number of “silent” synapses, in which only NMDARs are expressed at the postsynaptic site, is correlated with an AMPAR/NMDAR ratio less than one in hippocampal CA1 neurons (Isaac et al., 1995), it is intriguing to test whether proportionally there are less silent synapses on hypocretin neurons than on hippocampal CA1 neurons. If it is true, it is reasonable to postulate an interesting scenario. A small excitatory input mediated by glutamate may proportionally lead to more activated glutamatergic synapses on hypocretin neurons than on CA1 neurons, because silent synapses containing only NMDARs require a substantial depolarization to remove the Mg^2+^ blockade of NMDA receptors to conduct glutamatergic transmission (Isaac et al., 1995) and thus the synaptic transmission may “fail” at these synapses. Therefore, it is expected that glutamatergic transmission onto hypocretin neurons might be highly efficient without silent synapses.

Thirdly, GluR2-lacking calcium permeable AMPARs (Cp-AMPARs) are expressed in hypocretin neurons under basal conditions. The NMDAR receptor-mediated calcium influx is critical to synaptic plasticity (such as long-term potentiation (LTP)) in central neurons (Glasgow et al., 2015). Cp-AMPAR is recognized as a critical promoter to the development of synaptic plasticity in the brain as well. In central neurons where Cp-AMPARs are not expressed under basal conditions, such as pyramidal neurons in the CA1 region of the hippocampus, the initial and transient incorporation of Cp-AMPARs at postsynaptic sites during the induction phase is essential to the expression of NMDAR-dependent LTP (Plant et al., 2006). In central neurons where Cp-AMPARs are expressed under basal conditions, such as interneurons in the basolateral amygdala, the expression of LTP is NMDAR-independent and requires Cp-AMPARs (Mahanty and Sah, 1998). Our most recent studies have demonstrated the expression of Cp-AMPARs in hypocretin cells under baseline conditions (Rao et al., 2013). Although it has not been demonstrated, it is very likely that Cp-AMPARs may be responsible for calcium influx during glutamate-induced excitation of hypocretin cells and expression of synaptic plasticity in these cells.

**Figure 1:**
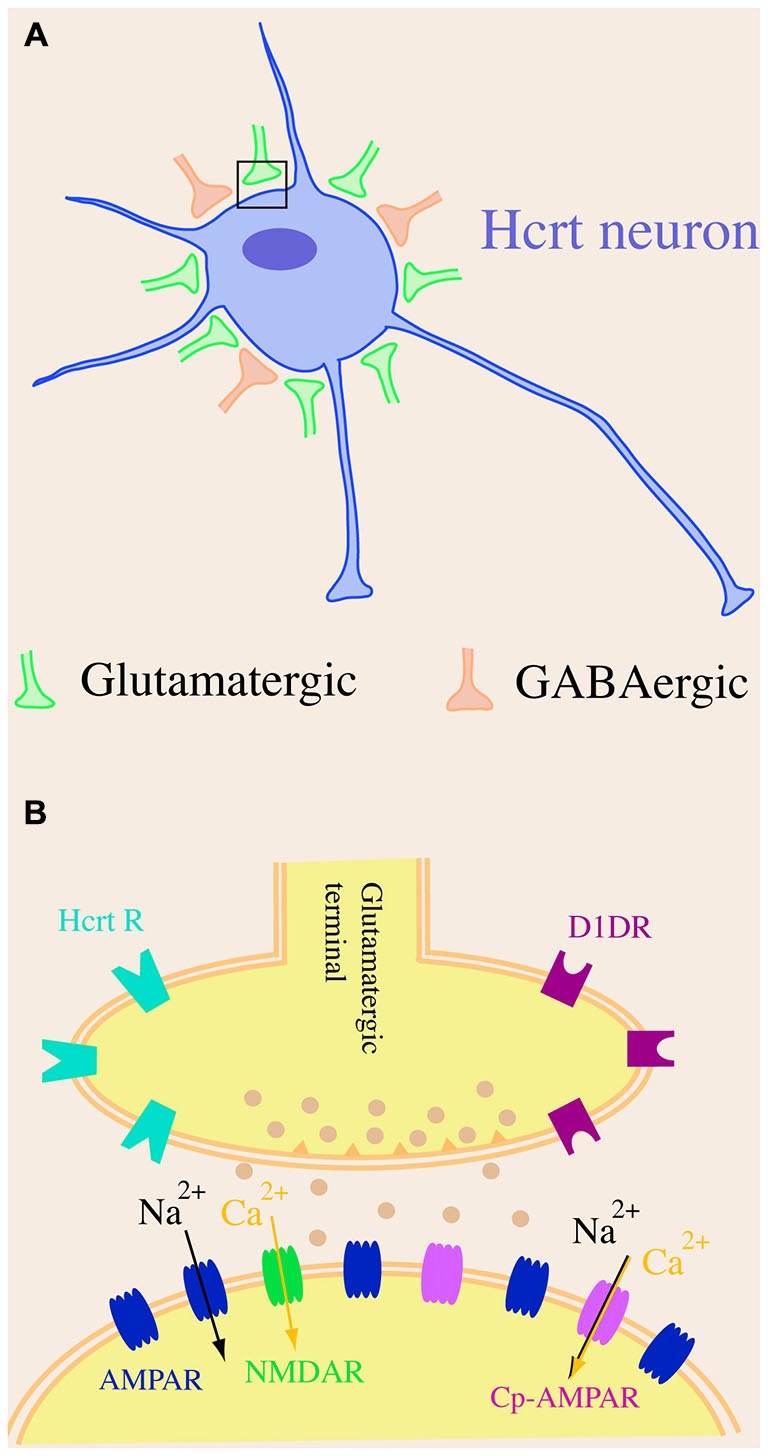
**A schematic diagram of synaptic organization in hypocretin neurons. (A)** Hypocretin-containing cell bodies are predominantly innervated by excitatory but not inhibitory synapses. There are more excitatory (glutamatergic) synapses than inhibitory (GABAergic) synapses on hypocretin-containing cell bodies. **(B)** The AMPA receptor (AMPAR)-carried excitatory postsynaptic current (EPSC) is significantly larger than NMDA receptor (NMDAR)-carried EPSC in hypocretin neurons, suggesting that a dominating number of AMPARs [including GluR2-lacking, calcium permeable AMPARs (Cp-AMPARs)] are expressed in hypocretin neurons.

Altogether, the unique glutamatergic and GABAergic synaptic organization on somata of hypocretin neurons as well as the composition of glutamate receptors on hypocretin cells (Figure 1) provide a functional basis for the role that these cells play in the regulation of the sleep/wake cycle, energy metabolism and other functions. The predominant innervation by excitatory synapses on cell bodies of hypocretin neurons and the highly efficient glutamatergic transmission are likely to facilitate the excitation of this system upon exposure to environmental cues and promote plasticity in response to physiological and environmental factors.

## Changes in Synaptic Efficacy in Hypocretin Neurons Induced by Daily Activities in Animals

Animals perform many daily activities, such as selecting habitats, foraging for food, and escaping from predators to sustain the survival of themselves and their species in the natural environment. The hypocretin system plays a central role in coordinating these daily functions. For instance, the levels of hypocretin in the cerebrospinal fluid are low during the resting phase and high during the active phase in animals and humans (Estabrooke et al., 2001; Blouin et al., 2013), which is consistent with the levels of electrical activity in hypocretin neurons recorded *in vivo* in freely moving or head-fixed rodents (Lee et al., 2005; Mileykovskiy et al., 2005). The activity in hypocretin neurons is also increased during the initial period of feeding and when animals are exposed to novel stimuli (Mileykovskiy et al., 2005). Therefore, it is reasonable to postulate that hypocretin neurons may undergo some plastic changes during intensive activation according to Hebbian theory.

### Neural Plasticity in Hypocretin Neurons in Energy Intake and Expenditure

Early evidence suggested that hypocretin was a feeding promoter when administered to animals *in vivo* (Sakurai et al., 1998). Food deprivation induced an increase in the expression of c-Fos in hypocretin neurons and hypocretin mRNA in animals (Sakurai et al., 1998; Diano et al., 2003). In animals with hypocretin deficiency, the anticipatory activity during food deprivation is attenuated and food deprivation does not induce wakefulness in animals (Yamanaka et al., 2003; Akiyama et al., 2004). These results have revealed a strong association between the arousal levels and feeding behaviors in animals, which is consistent with the well-established phenomenon that hunger induces arousal/wakefulness in animals (Jacobs and McGinty, 1971; Borbély, 1977). However, what remained unclear were the changes which occurred in the hypocretin system during food deprivation and how these changes might contribute to the regulation of feeding behaviors in animals. Our studies on the experience-dependent synaptic plasticity in hypocretin neurons, induced by food deprivation, provided new insights into the role of hypocretin in the link between energy homeostasis and arousal regulation (Horvath and Gao, 2005).

In C57/B6 mice undergoing one episode of fasting for 12 h or fed with normal chow, brain slices containing the Pf/LH area were prepared and whole-cell patch clamp recording was performed in hypocretin neurons expressing GFP under the control of a selective hypocretin promoter. The frequency of mEPSCs recorded in hypocretin neurons was significantly potentiated in fasted mice as compared with the fed littermates, while the frequency of mIPSCs recorded in these neurons was comparable between fasted and fed groups (Horvath and Gao, 2005). Consistent with electrophysiological results, ultrastructural investigations of hypocretin neurons demonstrated that the number of asymmetric (excitatory) but not symmetric (inhibitory) synapses on hypocretin-containing cell bodies was significantly increased in fasted mice as compared with fed controls (Horvath and Gao, 2005). It has been reported that in fasting animals the levels of leptin were low (Maffei et al., 1995), which may serve as a cue for food/energy deficiency in animals. Therefore, it was essential to test whether the lowered level of leptin triggered changes in fasting animals and whether the leptin replacement during food deprivation compensated for the effects triggered by fasting. Our data suggested that leptin replacement (i.p.) abolished the effects of fasting on glutamatergic synapses on hypocretin neurons in fasted mice. The frequency of mEPSCs was comparable in hypocretin neurons in fasted mice with leptin replacement during fasting and fed controls. The number of asymmetric synapses on hypocretinergic cell bodies was comparable between these two groups as well (Horvath and Gao, 2005). Consistent with the effect of leptin replacement on fasting-triggered synaptic plasticity in hypocretin neurons, electrophysiological and ultrastructural studies further showed that re-feeding reversed the effects of food deprivation on the frequency of mEPSCs and the number of asymmetric synapses on hypocretin cells in fasted mice (Horvath and Gao, 2005).

The physiological implications of plasticity in hypocretin neurons induced by food deprivation are not yet clear. At the cellular level, the enhanced excitatory inputs may significantly increase the activity in hypocretin neurons based on the unique synaptic architecture of these cells (Li et al., 2002; Horvath and Gao, 2005; Rao et al., 2013). At the whole animal level, the synaptic plasticity may be required to promote arousal or motivation to forage for food, although it has yet to be demonstrated. Many critical questions that may help establish the physiological significances and pathological implications of synaptic plasticity in hypocretin neurons in the context of food deficiency remain unanswered. Firstly, the origin of the synapses that are potentiated during food deprivation is not clear. Answers to this question will help address how complex foraging behavior is initiated. Secondly, factors that mediate and modulate the expression of neuroplasticity induced by food deprivation are not well established. We have shown the role of leptin, a metabolic cue, in the development of synaptic plasticity in hypocretin neurons (Horvath and Gao, 2005). The exact mechanisms underlying the role of leptin in the regulation of synaptic plasticity in hypocretin neurons are not yet clear since hypocretin neurons do not express leptin receptors (LepRbs; Louis et al., 2010; Goforth et al., 2014). Since the LepRb-expressing cells in the lateral hypothalamus directly innervate hypocretin cells (Louis et al., 2010; Goforth et al., 2014), a likely scenario is that the activation of LepRb-expressing cells may lessen the activity in hypocretin neurons to compromise the expression of synaptic plasticity. Further investigations are needed to establish the effects of leptin and other molecules encoding the energy state of animals on synaptic plasticity in hypocretin neurons. Thirdly, the intracellular mechanisms underlying the plasticity and the regulation of intracellular signaling pathways by metabolic cues need to be identified. Lastly, it is not clear how the synaptic plasticity induced by food intake is reversed by factors such as re-feeding. The excitation of hypocretin neurons is required to promote food intake (Sakurai et al., 1998), whereas it also promotes energy expenditure (Gao and Horvath, 2014; Zink et al., 2014). Therefore, the termination of the development of synaptic plasticity or de-potentiation of enhanced synaptic efficacy may be required to reduce energy expenditure and promote the store of energy in animals, which is particularly critical to animals in the natural environment.

### Synaptic Plasticity in Hypocretin Neurons in Sleep-Wake Homeostasis

It is well established that the hypocretin system is required to promote wakefulness and arousal and that the activity level of hypocretin neurons is high during wakefulness and low during sleep (Estabrooke et al., 2001; Lee et al., 2005; Mileykovskiy et al., 2005; Blouin et al., 2013). It is plausible to postulate that a long-term activation of hypocretin neurons is required in order to maintain a prolonged (voluntary or forced) wake state in animals. Therefore, hypocretin neurons are activated along with other glutamatergic neurons that innervate hypocretin cells to conduct information encoding environmental cues causing prolonged wakefulness. According to Hebbian theory, the efficacy of glutamatergic synapses on hypocretin neurons will be potentiated in an experience-dependent manner during the prolonged wakefulness.

To test our hypothesis, animals were kept in a state of prolonged wakefulness with two (chemical and physical) approaches. First, we applied a psychostimulant modafinil to animals during the rest (sleep) phase. Modafinil (diphenylmethyl-sulfony-2-acet-amide), an FDA-approved drug for the treatment of narcolepsy and other conditions (Ballon and Feifel, 2006), significantly enhances wakefulness in humans and animals through the activation of DA-dependent pathways and hypocretin neurons (Scammell et al., 2000; Wisor et al., 2001; Korotkova et al., 2007). An acute administration of a single dose of modafinil led to a long-lasting (>2 h) wakefulness in mice during the light (sleep) phase. The frequency and amplitude of mEPSCs and AMPAR/NMDAR ratio of evoked EPSCs were significantly increased 1 and 2 h after the administration of modafinil in hypocretin neurons from modafinil-treated mice as compared with control mice (with normal sleep), suggesting that synaptic potentiation might occur at both pre- and postsynaptic sites of glutamatergic synapses on hypocretin neurons (Rao et al., 2007). Next, we performed sleep deprivation for 4 h in mice with gentle handling, in which mice were kept awake by being gently touched with a small paintbrush upon the closure of their eyes by an experimenter (Modirrousta et al., 2005). Consistent with results from modafinil experiments, a sleep deprivation for 4 h induced a similar potentiation of glutamatergic synapses on hypocretin neurons at pre- and postsynaptic sites (Rao et al., 2007). The synaptic plasticity induced by prolonged wakefulness did not occur in neighboring non-hypocretin neurons, demonstrating the specificity of the effects of prolonged wakefulness (Rao et al., 2007).

To identify the mechanisms underlying the expression of synaptic potentiation induced by the prolonged wakefulness, D1 DA receptor antagonists were applied to animals before the administration of modafinil. D1 antagonists abolished modafinil-induced wake-promoting effects and the potentiation of presynaptic glutamate release in animals, demonstrating the requirement of DA-mediated pathways in this process (Rao et al., 2007). This is also consistent with the report that the action of modafinil depends on the blockade of DA transporters in the brain (Ballon and Feifel, 2006) and that DA receptor-mediated pathways modulate activity in hypocretin neurons (Bubser et al., 2005).

Next, we tested whether the expression of LTP was occluded in hypocretin neurons undergoing modafinil treatment. It has been shown that forskolin-induced activation of protein kinase A (PKA) induces LTP (for-LTP) in hippocampal neurons and that for-LTP occludes (prevents) the induction of LTP triggered by other stimuli (such as high frequency stimulation) at the same synapses (Frey et al., 1993; Huang and Kandel, 1995; Otmakhov et al., 2004). These results suggest that for-LTP shares the same pathways with LTP induced by high frequency stimulation in the hippocampus. Our data showed that for-LTP was induced at glutamatergic synapses on hypocretin neurons both pre- and postsynaptically (Rao et al., 2007). Consistent with our hypothesis, the induction of for-LTP was significantly occluded in hypocretin neurons in mice acutely or repeatedly exposed to modafinil, indicating that synaptic plasticity induced by modafinil treatment shared common pathways (e.g., PKA-mediated pathways) with for-LTP in these cells (Rao et al., 2007). Therefore, the D1 DA receptor-PKA pathway may be required in the expression of synaptic plasticity in hypocretin neurons.

The functional consequences of experience-dependent plasticity in hypocretin neurons are not yet clear. It is hypothesized that one of the functions of sleep is to rejuvenate the nervous system by depressing or de-potentiating the enhanced synaptic efficacy in central neurons during the wake phase (Tononi and Cirelli, 2006, 2014). The enhanced synaptic efficacy in hypocretin neurons by prolonged wakefulness provides the first piece of evidence that wakefulness may enhance synaptic efficacy in central neurons. Later we showed similar results in cortical neurons that synaptic efficacy potentiated and de-potentiated in cortical neurons across the natural transition from wake to sleep (Liu et al., 2010). Our data suggest that synaptic plasticity in hypocretin neurons induced by prolonged wakefulness may be activity-dependent, which makes it reasonable to postulate that the enhanced synaptic efficacy is the consequence of the prolonged wakefulness. Therefore, we have proposed that synaptic potentiation in hypocretin neurons may be required to maintain prolonged wakefulness in animals and that the development of synaptic plasticity in hypocretin neurons may provide a mechanism through which the arousal threshold is regulated to determine the behavioral state of animals (Gao and Wang, 2010). This hypothesis has not been demonstrated in mammals, but results from studies on structural plasticity of synapses on hypocretin neurons in zebra fish provide a clear first step in this direction (Appelbaum et al., 2010).

### Convergence of Energy Balance and Sleep-Wake Regulation in Hypocretin Neurons

The energy hypothesis of sleep proposes that one of the functions of sleep is to preserve energy, through which animals may cope with the natural environment where food is not always available due to the cyclic transition between the dark and light phases. As a potent arousal promoter, the hypocretin system is under the regulation of many molecules encoding ambient energy supplies and energy use such as glucose, lactate, dietary amino acids, and adenosine (Yamanaka et al., 2003; Burdakov et al., 2006; Liu and Gao, 2007; Xia et al., 2009; Parsons and Hirasawa, 2010; Karnani et al., 2011; Liu et al., 2011). The regulation of the activities in hypocretin neurons by adenosine is particularly critical to establish the importance of these neurons for regulation of energy states and behavioral/arousal status. On the one hand, adenosine is a potent sleep-promoting substance (Porkka-Heiskanen and Kalinchuk, 2011). However, the extracellular levels of adenosine are closely relevant to the energy metabolism within nerve cells (Porkka-Heiskanen and Kalinchuk, 2011). Therefore, the utilization of energy can be translated into a mechanism used by the hypocretin system as a cue to limit energy expenditure and modulate behavioral state. Consistently, recent data from us and others have shown that the intracellular levels of ATP play a key role in the maintenance of the membrane potential and generation of action potentials in these neurons (Parsons and Hirasawa, 2010; Liu et al., 2011) and that the intracellular ATP levels in hypocretin neurons are lower in the sleep state than in the sleep-deprived state in animals (Liu et al., 2011). These results, along with other evidence, may help explain the mechanisms underlying the findings that the energy status of animals is highly correlated with their behavioral state (Shulman et al., 2003). For example, the brain utilizes more energy in the aroused state than in the quiet anesthetized state and a low energy state leads to unconsciousness in animals (Shulman et al., 1999, 2009).

The role of neural plasticity in hypocretin neurons in the determination of behavioral states based on the energy state in animals is not clear. It has been well established that the brain utilizes a relatively large proportion of energy despite accounting for only a small portion of the animal body. It has been estimated that the maintenance of neurotransmission is extremely energy-consuming (Khatri and Man, 2013). It is not yet clear whether the expression of experience-dependent neural plasticity is altered in hypocretin neurons in animals under different energy states (such as calorie restriction and obesity), through which acute and chronic changes in energy balance may shape homeostatic and cognitive functions through the hypocretin system in animals and lead to various mental or psychiatric diseases in humans.

**Figure 2:**
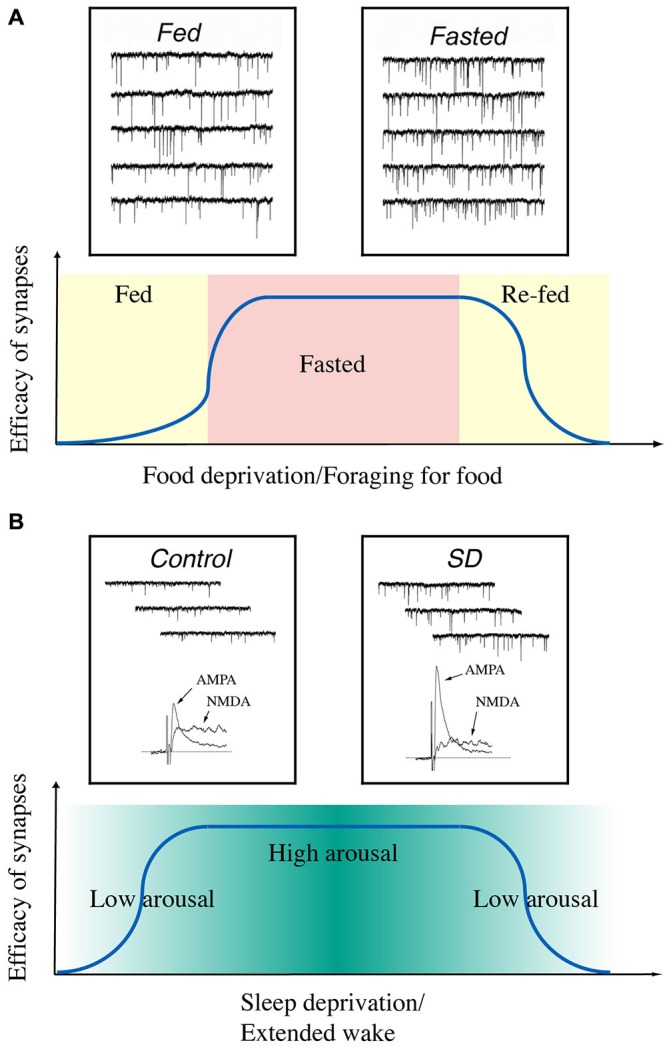
**A diagram summarizes the time courses of changes in synaptic efficacy of glutamatergic synapses on hypocretin neurons depending on the energy and arousal states in animals. (A)** Synaptic efficacy is potentiated when animals are food-deprived and de-potentiated when they are fed or re-fed. Insets, miniature excitatory postsynaptic currents (mEPSCs) recorded in hypocretin neurons in fed (left) and fasted (right) mice. Traces are from Horvath and Gao (2005) with permission from Cell Metabolism. **(B)** Synaptic efficacy is potentiated when the arousal levels are high (sleep deprived) in animals and de-potentiated when they are sleeping. Insets, mEPSCs and AMPAR- and NMDAR-carried EPSCs recorded in hypocretin neurons from sleeping (left) and sleep-deprived (right) mice. Traces are from Rao et al. (2007) with permission from the Journal of Clinical Investigation.

In summary, our current data indicate that the synaptic efficacy of glutamatergic synapses on hypocretin neurons is enhanced when animals are hungry and when their arousal levels are high (Figure 2). It may suggest that the fluctuations in the energy states and arousal states may lead to changes in synaptic efficacy (synaptic plasticity) in hypocretin neurons on a daily basis in animals. Therefore, synaptic plasticity induced by daily functions of hypocretin neurons may be more complicated than we discussed here, since many factors (such as the drives to forge for food and stay alert to avoid predators) may exert their effects on hypocretin neurons simultaneously. It is not yet clear whether these factors may generate synergistic or antagonistic effects on hypocretin neurons. Studies along this line will provide new avenues to address how the neuronal circuitry centered on hypocretin neurons may be fine-tuned to execute their daily functions in animals.

## Neural Plasticity in the Hypocretin System Induced by Long-Term Behavioral Changes in Animals

The changes in synaptic efficacy in hypocretin neurons resulting from daily activities of animals may be essential for hypocretin neurons to receive inputs encoding cues about the internal and external environments of animals, make “decisions” and send out outputs to the downstream effector targets on a daily basis. Most recent data from others and us further show that in animals experiencing and accommodating long-term changes in the internal and external environments the hypocretin system may undergo plasticity as the consequence of adaptation or maladaptation.

### Synaptic Plasticity in Hypocretin Neurons in Animals Chronically Exposed to Wake-Promoting Psycho-Stimulants

The effects of long-term sleep loss on brain functions, particularly neuronal plasticity, have been intensively investigated in brain regions highly relevant to learning/memory and other cognitive functions (Kreutzmann et al., 2015). Since long-term sleep loss is a critical risk factor that may lead to metabolic disorders such as obesity and diabetes and the hypocretin system is a critical player in the regulation of metabolic functions of the brain (Copinschi et al., 2014; St-Onge and Shechter, 2014), we examined the effects of a chronic exposure to reduced sleep on the hypocretin system in mice through applying modafinil daily to mice during the light phase for a week (Rao et al., 2007). The application of modafinil increased activities (reduced sleep) during the light phase in animals every day throughout the regimen (Rao et al., 2007). One day after the completion of chronic treatment, we found that the number of asymmetric (excitatory) synapses on hypocretin neurons was significantly elevated in addition to the enhancement in the frequency and amplitude of mEPSCs in these cells. In addition, the potentiation of glutamatergic synapses on hypocretin neurons by the activation of PKA was occluded in mice chronically exposed to modafinil-induced sleep reduction, suggesting the involvement of PKA-mediated pathways in changes in synaptic efficacy in hypocretin neurons induced by a chronic sleep loss. These results suggest that chronic sleep loss may cause long-term effects on the wake-promoting hypocretin neurons (Rao et al., 2007).

This study may have many implications for our understanding of the impacts of long-term sleep loss on the homeostatic functions of the brain. Firstly, it is not clear whether the plastic changes in hypocretin neurons and other neurons induced by long-term sleep loss are reversible or not under normal physiological conditions or therapeutic interventions. In our previous study (Rao et al., 2007), we have not explored the time course of synaptic changes in hypocretin neurons after the termination of chronic sleep restriction. Secondly, it is essential to understand whether the changes induced by long-term sleep loss may have long-term impacts on sleep regulation in animals. Although direct evidence along this direction is lacking, current results have implied this possibility. A chronic sleep deprivation was reported to be able to cause allostatic changes in animals, in which repeated sleep deprivation did not lead to enhanced sleep intensity in animals (Kim et al., 2007). A later report challenged the allostatic changes in the brain during repeated sleep deprivation (Leemburg et al., 2010). However, the authors in the later study did report compromised homeostatic responses to chronic sleep deprivation in certain brain areas (Leemburg et al., 2010). Therefore, it is reasonable to hypothesize that some changes occurring in certain brain areas during an acute sleep loss may accumulate over the time course of chronic sleep loss to cause allostatic responses reported by Kim et al. (2007) while similar changes in other brain areas may be reversible after a recovery sleep so that chronic sleep loss would not induce allostatic responses in these areas (Leemburg et al., 2010). It is essential to examine the mechanisms underlying the allostatic changes induced by chronic sleep loss and test the hypothesis whether neuronal plasticity in wake-promoting systems such as the hypocretin system is involved in these changes. Thirdly, it is essential to understand whether the changes induced by long-term sleep loss may have long-term impacts on other homeostatic regulations such as the regulation of energy intake and expenditure. It is well known that sleep loss is a risk factor for the development of obesity and diabetes (Copinschi et al., 2014; Cedernaes et al., 2015). It is not yet clear to what extent the neuroplasticity in hypocretin neurons induced by long-term changes in the sleep/wake cycle may contribute to the altered energy metabolism in animal models and human patients. However, strong implications of the role of hypocretin in the development of metabolic diseases resulting from chronic sleep loss are emerging (Nixon et al., 2015).

### Synaptic Plasticity in Hypocretin Neurons in Animal Models of Drug Addiction

The early observations have established the Pf/LH area as a brain structure involved in reward-seeking behaviors in animals. The electrical stimulation of this brain region acutely induced a profound reinforcement activity and a robust self-administration in rodents (Olds and Milner, 1954; Olds, 1958). Addictive drugs (such as morphine and amphetamine) can modulate the self-administration of electrical stimulation to the Pf/LH in these animals (Adams et al., 1972; Goodall and Carey, 1975). Moreover, addictive drugs induced marked self-reinforcing effects in animals when they were directly administered into the Pf/LH area (Olds and Williams, 1980; Cazala et al., 1987). Revealing of the role of the hypocretin system in mediating reward-seeking behaviors has opened a new chapter for the study of the Pf/LH area.

It is now clear that hypocretin neurons are required for the development of reward seeking and addiction in animal models and human patients (see reviews by Baimel and Borgland, 2012; España, 2012; Mahler et al., 2012). Firstly, hypocretin neurons are activated (measured by an increase in the expression of c-fos) when animals are exposed to opiates, cocaine, amphetamine and nicotine in several animal models of drug seeking behaviors (Georgescu et al., 2003; Harris et al., 2005; Pasumarthi et al., 2006; McPherson et al., 2007; Plaza-Zabala et al., 2011). Secondly, direct infusion of hypocretin into reward centers in the brain or activation of hypocretin neurons promotes drug seeking in animals (Boutrel et al., 2005; Harris et al., 2005; Hamlin et al., 2008; España et al., 2011). Thirdly, disruption of hypocretin receptors-mediated signaling with pharmacological and genetic approaches attenuates or blocks drug-seeking behaviors in animals (Georgescu et al., 2003; Harris et al., 2005; Borgland et al., 2006, 2009; Hollander et al., 2008, 2012; LeSage et al., 2010; España et al., 2011). In humans, narcoleptic patients with a deficiency in hypocretin peptide or hypocretin neurons exhibit a lowered tendency to drug abuse (Guilleminault et al., 1974).

According to the current framework, drug-induced plasticity in reward circuitry in the brain is a critical mechanism underlying the development of drug addiction (Hyman et al., 2006; Kalivas, 2007; Lüscher and Malenka, 2011). Hypocretin is required in the expression of synaptic plasticity in reward centers (such as the VTA) induced by addictive drugs (Borgland et al., 2006; Winrow et al., 2010). The latest evidence from others and us has shown that the hypocretin system undergoes synaptic plasticity in animals exposed to drugs of abuse. In rats self-administering cocaine or being treated with cocaine by experimenters for seven days, the efficacy of glutamatergic synapses on unidentified perifornical/LH neurons is enhanced, as demonstrated by an increased frequency but not amplitude of miniature EPSCs (Yeoh et al., 2012). The authors did not find changes in the AMPAR/NMDAR ratio, but found a paired-pulse depression (PPD) in Pf/LH neurons in cocaine-exposed rats, suggesting that synaptic plasticity in glutamatergic synapses on these neurons may be presynaptic in nature (Yeoh et al., 2012). By using immunocytochemical methods, the authors reported that the numbers of VGLUT2-positive puncta closely apposed to hypocretin neurons were increased and that the frequency of mEPSCs recorded in neurobiotin-labeled hypocretin cells was enhanced in cocaine-treated rats as compared to controls (Yeoh et al., 2012). This is the first piece of evidence that cocaine exposure may induce neuroplasticity in the Pf/LH area and hypocretin neurons may be among nerve cells that undergo plastic changes in animals exposed to addictive drugs.

**Figure 3:**
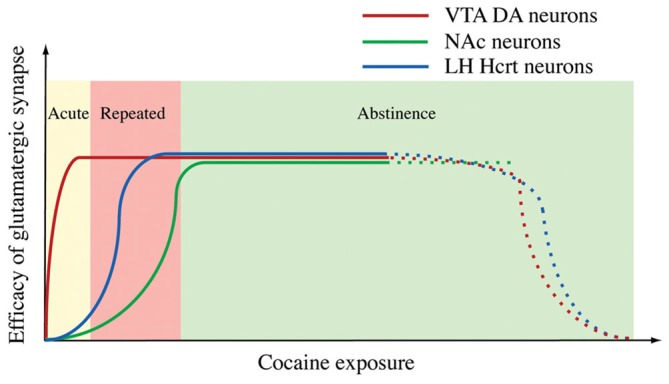
**A diagram summarizes the time courses of synaptic potentiation in the ventral tegmental area (VTA) (Ungless et al., 2001), NAc (Kourrich et al., 2007) and Hcrt neurons induced by exposure to cocaine.** From Rao et al. (2013) with permission from Journal of Physiology.

We visited this issue by directly examining changes in GFP-labeled hypocretin neurons in mice. Our results indicated that after the establishment of cocaine conditioned place preference (CPP), the amplitude, but not the frequency of mEPSCs, was enhanced in hypocretin neurons and that the AMPAR/NMDAR ratio was increased as well in these cells (Rao et al., 2013). These results suggest changes in the postsynaptic site of glutamatergic synapses on hypocretin neurons, which is consistent with the observations made in DA neurons in the VTA and NAc (Ungless et al., 2001; Kourrich et al., 2007). When a selective HcrtR1 antagonist SB334867 was applied to block the development of cocaine CPP in mice, the synaptic plasticity in hypocretin neurons still existed (Rao et al., 2013). We also examined the time course of the expression of cocaine-induced plasticity in hypocretin neurons. In contrast to synaptic potentiation in DA neurons induced by an acute (single) injection of cocaine in the VTA (Ungless et al., 2001), the same treatment was not sufficient to potentiate glutamatergic synapses on hypocretin neurons. In contrast to the report on the expression of synaptic potentiation in DA neurons during the withdrawal of cocaine in the NAc (Kourrich et al., 2007), the synaptic potentiation in hypocretin neurons after a short-term (three days) cocaine exposure lasted for at least five days, but not longer than ten days during withdrawal (Rao et al., 2013). These results suggest that the expression of synaptic plasticity in hypocretin neurons may be at an intermediate stage of the development of behaviors relevant to addiction in animals (Figure 3). We also showed that synaptic plasticity induced by a short-term cocaine exposure did not occlude but facilitated the expression of tetanic stimulation-induced LTP (HFS-LTP) in hypocretin neurons, providing interesting functional implications. Since HFS-induced LTP is expressed at both pre- and postsynaptic sites of synapses while LTP induced by cocaine exposure occurs at the postsynaptic site of synapses on hypocretin neurons, the facilitation of HFS-LTP in hypocretin neurons in cocaine-treated mice is consistent with our current understanding of synaptic plasticity. This result suggests that the same presynaptic release of glutamate may induce a greater synaptic response in hypocretin neurons due to the enhanced postsynaptic response. Therefore, it may lower the threshold for the induction of LTP by subsequent stimulations. From a systems point of view, the synaptic plasticity induced by chronic cocaine treatments is a type of metaplasticity in these glutamatergic synapses, which may allow them to integrate many other inputs encoding environmental cues (such as stress) that activate them to form a strong association with the cocaine experience. This may explain why hypocretin neurons are involved in stress-induced, but not context-elicited, drug-seeking behavior (Boutrel et al., 2005; Aston-Jones et al., 2010).

It is apparent that many factors may contribute to the discrepancies existing in the current reports on neural plasticity in hypocretin neurons in animals exposed to cocaine (Yeoh et al., 2012; Rao et al., 2013). Firstly, different species were used in these reports, with rats in the study by Yeoh et al. (2012) and mice in that by Rao et al. (2013). Secondly, the animal models of drug seeking and time courses of drug exposure were distinct in these studies. Yeoh et al. (2012) used cocaine self-administration and a time course consistent with the development of self-administration, while Rao et al. (2013) used CPP and a time course relevant to the expression of CPP and withdrawal. Thirdly, the results from Yeoh et al. (2012) included non-identified Pf/LH neurons and neurobiotin-labeled hypocretin neurons. In the report by Rao et al. (2013), the observations were directly and solely made in hypocretin neurons in transgenic mice expressing GFP specifically in these cells. Therefore, the discrepancy in the specificity of cell types involved in these two studies may contribute profoundly to the reported results. In addition, even the hypocretin neurons *per se* may be heterogeneous in mediating reward-seeking behaviors (Harris and Aston-Jones, 2006), which may contribute additional complexity to the understanding of drug-induced plasticity in hypocretin neurons. Future studies, building on these early findings, using new approaches such as optogenetics and DREADDs (Designer Receptors Exclusively Activated by Designer Drugs) are merited, in which the specificity of the roles of hypocretin neuron subtypes in mediating reward-seeking behaviors could be examined.

### The Role of Hypocretin in Behavioral Changes in Animals Exposed to Abused Drugs

A growing body of evidence has emerged on the participation of hypocretin in reward seeking and motivational behaviors in animals and humans. The hypocretin system may be responsible for cue-induced seeking for cocaine and morphine (Georgescu et al., 2003; Harris et al., 2005). The hypocretin system may also play a critical role in the motivational aspect of drug-seeking behavior, as shown in cocaine self-administration experiments (Borgland et al., 2009). It was recently shown that hypocretin activates neurons in the ventral pallidum (VP) to generate a hedonic (liking) response to sweetness in rats (Ho and Berridge, 2013). Therefore, it is reasonable to postulate that the hypocretin system may participate in every stage of the development of reward seeking and addictive behaviors, from the sensing of reinforcers to produce hedonic responses in the brain, to the establishment of associations between environmental cues and the reinforcers and ultimately to the development of motivation to seek the reinforcers. It is conceivable that the functional and structural connections between the hypocretin system and other brain centers involved in the generation of motivational behaviors would be strengthened during the process. Since it has been well established that synaptic plasticity is a powerful mechanism for the CNS to re-wire itself in order to accommodate behavioral changes in animals, the plasticity in the hypocretin system is expected to play a central role in the formation of reward-seeking and motivational behaviors mediated by the Pf/LH area. Currently it is not clear whether synaptic plasticity in hypocretin neurons is required in the development of addictive behaviors in animals.

Drug abuse has led to many other health issues in addition to addictive behaviors in humans. It has been reported that patients abusing illicit drugs suffer from significant sleep loss and that the extent of sleep loss may be an indicator for the relapse to drug abuse (Morgan and Malison, 2007; Valladares and Irwin, 2007; Brower and Perron, 2010). The hypocretin system is at the crossroad of the regulation of sleep homeostasis and drug abuse, making it a potential target for addressing sleep disorders observed in patients abusing psycho-stimulants. As a part of the wakefulness-promoting circuitry in the brain, the activity in hypocretin neurons is closely associated with the arousal state of the animal (Estabrooke et al., 2001; Zeitzer et al., 2003; Lee et al., 2005; Mileykovskiy et al., 2005; Adamantidis et al., 2007). We have shown in a mouse model with deficiency in the receptor for melanin-concentrating hormone (MCH) that the activation of Hcrt neurons is facilitated due to the up-regulation of synaptic efficacy in glutamatergic transmission onto these cells, which likely contributes to a lowered wake-promoting threshold in these mice (Rao et al., 2008). Although it has not been demonstrated in mammals, an enhancement of the number of synapses on hypocretin neurons through genetic manipulations significantly attenuates sleep promotion in zebrafish (Appelbaum et al., 2010). Therefore, it is likely that the potentiation of glutamatergic synapses on hypocretin neurons may contribute to impaired sleep in drug-addicted patients.

## Clinical Implications of Neural Plasticity in the Hypocretin System

As a definitive regulator of appetite, sleep, energy balance, and reward, the hypocretin system likely plays a crucial role in the development of psychiatric conditions and mental diseases. Neurovegetative symptoms, including disruptions in sleep, appetite, motivation and reward, are core features of nearly every major mental and neurological illness. The deficiency in the hypocretin system has been reported in many neurological diseases. The specific role that hypocretin plays in narcolepsy described in the DSM-V (Diagnostic and Statistical Manual of Mental Disorders, fifth edition), as a disorder in which the individual will experience recurrent periods of an irresistible need to sleep, has now been established, both in preclinical models and through human studies (Chemelli et al., 1999; Lin et al., 1999; Nishino et al., 2000; Thannickal et al., 2000; Ripley et al., 2001). In animal models, the loss of the hypocretin peptide, its receptors (particularly OX2R) or hypocretin-containing neurons results in a narcolepsy-like phenotype (Chemelli et al., 1999; Lin et al., 1999; Hara et al., 2001; Yamanaka et al., 2003; Willie et al., 2003). In human cases of narcolepsy the cause of a low (or undetectable) level of hypocretin in the CSF of patients with narcolepsy–cataplexy is likely due to the loss of Hcrt-containing neurons but not hypocretin peptide or receptor genes (Thannickal et al., 2000). An autoimmune process has been proposed to be responsible for this pathological condition (Han, 2012; Mahlios et al., 2013). The replacement of hypocretin is currently under preclinical development as a potential therapeutic treatment for narcolepsy in narcoleptic animal models (Blanco-Centurion et al., 2013; Kantor et al., 2013). The loss of hypocretin neurons has also been reported in Parkinson’s disease (PD) and correlated with the progress of the disease (Fronczek et al., 2007; Thannickal et al., 2007). In addition to PD, the loss of the hypocretin neurons in the LH area is also identified in the brains of patients with Alzheimer’s disease (AD; Fronczek et al., 2012), post-traumatic injury (Baumann et al., 2009), and Dementia with Lewy bodies (DLB; Kasanuki et al., 2014). In a rare genetic disease Prader-Willi syndrome (PWS), caused by a deletion in the paternal chromosome 15 or by maternal uniparental disomy and characterized by weak muscle tone (hypotonia), poor growth, delayed development and chronic overeating, an impaired level of hypocretin-1 in the CSF is reported, which correlates with the severity of excessive daytime sleepiness (EDS) in these patients (Nevsimalova et al., 2005). Since the total number of hypocretin neurons is not significantly different in PWS patients as compared to age-matched controls (Fronczek et al., 2005), it is possible that the reduced CSF levels of hypocretin may result from an impaired functionality of neurons synthesizing this neuropeptide in PWS patients. To date, there is little evidence on the role of neuroplasticity in hypocretin neurons in the diseases and conditions summarized above. However, data from animal studies may provide valuable insights into the development of new strategies to treat and manage these diseases. For example, if neuroplasticity in hypocretin neurons is able to enhance the functionality of these cells to enhance arousal levels as suggested by current data (Rao et al., 2007; Appelbaum et al., 2010), therapeutic interventions leading to gain-of-function modifications of hypocretin neurons would greatly improve the symptoms and the quality of lives of patients suffering from the deficiency of the hypocretin system.

Sleep disturbance is associated with many psychiatric and mental diseases. We know insomnia co-occurs in 20–40% of individuals with a mental illness (Ohayon, 2002; Soehner et al., 2013). Insomnia precedes the onset of depression symptoms and can also be used to predict relapse of this mood disorder (Breslau et al., 1996; Soehner et al., 2013). Disruptions in the sleep/wake cycle contribute to the prodromal state in early stages of psychosis and may in fact serve as an early biomarker of the disease. Sleep disturbance is also considered an early biomarker for bipolar disorder (BD); one study suggests that it is frequently the first symptom to emerge in the clinical expression of the disease (Zeschel et al., 2013). In the depressed phase of BD rates of insomnia have been reported to be 100%, while rates of hypersomnia or excessive sleep have been reported to range from 23% to 78%. A recent meta-analysis suggested that in patients with psychiatric diagnoses—depression, panic disorder, schizophrenia, and post-traumatic stress disorder (PTSD)—sleep disturbances are associated with the increased risk of suicidal behaviors (Malik et al., 2014). Despite the availability of sedative hypnotics, problems with insomnia persist for individuals across the spectrum of mental illness. In individuals with chronic, severe insomnia, there is a trend to develop resistance to long-term hypnotic treatment (Takaesu et al., 2014). The hypocretin system has been identified as an effective target for drug development and treatment of insomnia (Malik et al., 2014). The dual Hcrt receptor antagonist suvorexant has been shown to be effective as a treatment for insomnia in clinical trials (Herring et al., 2014; Winrow and Renger, 2014), providing a new path for understanding the natural history of insomnia and the role of the hypocretin system in this disorder. Most importantly, the potential involvement of the hypocretin system in the development of insomnia and other sleep disorders may provide a new avenue for the understanding of the roles of hypocretin and the LH area in endophenotypes of mental illness.

## Conclusions and Future Directions

**Figure 4:**
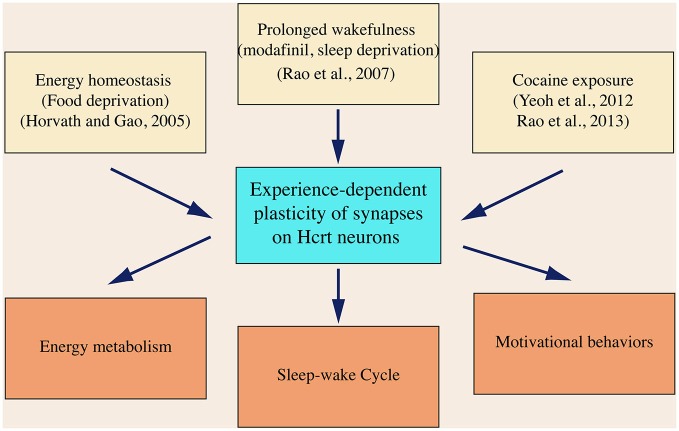
**A diagram summarizes the expression of synaptic plasticity in hypocretin neurons in animals exposed to various physiological and environmental factors.** In this diagram, it is also hypothesized that neural plasticity in hypocretin neurons is required to promote the brain functions governed by the hypocretin system, which has not been demonstrated so far.

Since it was discovered in 1998, the hypocretin system has emerged as a critical component in the brain circuitry, integrating neuronal signals encoding sensorimotor, environmental and homeostatic cues. Current data based on studies on hypocretin neurons from others and us support a concept that neural plasticity occurs in hypocretin neurons as the consequence of an intensive activation of these cells. As summarized in Figure 4, neuronal activities representing energy state (food deprivation), behavioral state (prolonged wakefulness) and behavioral change (drug seeking) trigger neuroplasticity in hypocretin neurons. Synaptic plasticity developed in these processes is likely required in mediating these behaviors by the hypocretin system, as discussed in the previous sections.

The experience-dependent neural plasticity has been established as a neurobiological mechanism underlying the learning and memory in animals. These plastic changes occurring in the cortex and hippocampus may be the biological basis of the memory trace (Bliss et al., 2013; Takeuchi et al., 2013). The role of experience-dependent plasticity in the regulation of homeostatic functions of the brain is less clear. Depending on the specific neuronal types involved in the synaptic plasticity, the functional connections between each component of the circuits may be strengthened or weakened, which would lead to an altered output of the circuits. As suggested by the studies on synaptic plasticity in hypocretin neurons and other neuronal types in the hypothalamus, neural plasticity may serve as a mechanism that leads to re-wiring of neural circuits responsible for the homeostatic regulations in animals. Since hypothalamic structures may also be involved in the regulation of complex behaviors (such as cognitive functions), as suggested in studies that have emerged recently, the neural plasticity in these brain areas may not serve as a memory trace but rather as a “behavioral trace”. A memory trace is retrievable upon exposure to cues leading to the specific memory (no matter whether it is explicit or implicit memory). The “behavioral trace”, which is not retrievable upon exposure to the same cues, yet may temporarily or permanently alter the affected neuronal circuits and re-shape the homeostatic and cognitive functions of the brain. Direct evidence supporting this hypothesis is not available currently, but studies on neural plasticity in hypocretin neurons and other hypothalamic systems, have shown this possibility.

In summary, neural plasticity is induced in hypocretin neurons by various physiological and environmental factors. The studies on plastic changes in hypocretin neurons are essential to the understanding of the roles of these cells in the control of homeostatic and cognitive functions in animals and the development of mental and psychiatric disorders in humans.

## Conflict of Interest Statement

The authors declare that the research was conducted in the absence of any commercial or financial relationships that could be construed as a potential conflict of interest.
